# Expression Analysis of Long Non-Coding RNAs Related With FOXM1, GATA3, FOXA1 and ESR1 in Breast Tissues

**DOI:** 10.3389/fonc.2021.671418

**Published:** 2021-05-19

**Authors:** Bita Hassani, Mohammad Taheri, Yazdan Asgari, Ali Zekri, Ali Sattari, Soudeh Ghafouri-Fard, Farkhondeh Pouresmaeili

**Affiliations:** ^1^ Department of Medical Genetics, Shahid Beheshti University of Medical Sciences, Tehran, Iran; ^2^ Urology and Nephrology Research Center, Shahid Beheshti University of Medical Sciences, Tehran, Iran; ^3^ Department of Medical Biotechnology, School of Advanced Technologies in Medicine, Tehran University of Medical Sciences, Tehran, Iran; ^4^ Department of Medical Genetics and Molecular biology, Faculty of Medicine, Iran University of Medical Sciences, Tehran, Iran; ^5^ Men’s Health and Reproductive Health Research Center, Shahid Beheshti University of Medical Sciences, Tehran, Iran

**Keywords:** breast cancer, lncRNAs, FOXM1/GATA3/FOXA1/ESR1 axis, APTR, AC144450.1, linc00663, ZNF337.AS1, RAMP2.AS1

## Abstract

Breast cancer is the most common neoplasm among females. Estrogen receptor (ESR) signaling has a prominent impact in the pathogenesis of breast cancer. Among the transcription factors associated with ESR signaling, FOXM1, GATA3, FOXA1 and ESR1 have been suggested as a candidate in the pathogenesis of this neoplasm. In the current project, we have designed an *in silico* approach to find long non-coding RNAs (lncRNAs) that regulate these transcription factors. Then, we used clinical samples to carry out validation of our in silico findings. Our systems biology method led to the identification of *APTR*, *AC144450.1*, *linc00663*, *ZNF337.AS1*, and *RAMP2.AS1* lncRNAs. Subsequently, we assessed the expression of these genes in breast cancer tissues compared with the adjacent non-cancerous tissues (ANCTs). Expression of *GATA3* was significantly higher in breast cancer tissues compared with ANCTs (Ratio of mean expressions (RME) = 4.99, P value = 3.12E−04). Moreover, expression levels of *APTR*, *AC144450.1*, and *ZNF337.AS1* were elevated in breast cancer tissues compared with control tissues (RME = 2.27, P value = 5.40E−03; Ratio of mean expressions = 615.95, P value = 7.39E−19 and RME = 1.78, P value = 3.40E−02, respectively). On the other hand, the expression of *RAMP2.AS1* was lower in breast cancer tissues than controls (RME = 0.31, P value = 1.87E−03). Expression levels of *FOXA1*, *ESR1*, and *FOXM1* and *linc00663* were not significantly different between the two sets of samples. Expression of *GATA3* was significantly associated with stage (P value = 4.77E−02). Moreover, expressions of *FOXA1* and *RAMP2.AS1* were associated with the mitotic rate (P values = 2.18E−02 and 1.77E−02, respectively). Finally, expressions of *FOXM1* and *ZNF337.AS1* were associated with breastfeeding duration (P values = 3.88E−02 and 4.33E−02, respectively). Based on the area under receiver operating characteristics curves, *AC144450.1* had the optimal diagnostic power in differentiating between cancerous and non-cancerous tissues (AUC = 0.95, Sensitivity = 0.90, Specificity = 0.96). The combination of expression levels of all genes slightly increased the diagnostic power (AUC = 0.96). While there were several significant pairwise correlations between expression levels of genes in non-tumoral tissues, the most robust correlation was identified between *linc00663* and *RAMP2.AS1* (r = 0.61, P value = 3.08E−8). In the breast cancer tissues, the strongest correlations were reported between *FOXM1*/*ZNF337.AS1* and *FOXM1*/*RAMP2.AS1* pairs (r = 0.51, P value = 4.79E−5 and r = 0.51, P value = 6.39E−5, respectively). The current investigation suggests future assessment of the functional role of *APTR*, *AC144450.1* and *ZNF337.AS1* in the development of breast neoplasms.

## Introduction

Breast cancer is the most frequent cancer and one of the leading causes of mortality in women (1). More than two-thirds of breast cancers are classified as luminal A/B subtypes according to the expression of the estrogen receptor α (ER-α) protein (2). ER-positive status has been regarded as an indicator of suitable patients’ prognosis. Yet, the emergence of resistance to endocrine therapy results in disease progression and mortality in many of these patients highlighting the importance of early diagnosis and establishment of effective treatment modalities for these patients (3). In response to estrogen, ER acts as a transcription factor to influence expression of several genes. However, several cofactors are involved in the process of ER-mediated gene expression regulation. These factors mainly modulate chromatin structure to make the compacted DNA available for ER to bind and exert its function (4). Moreover, some proteins construct scaffolds for additional crucial factors and several cofactors with enzymatic activities necessary for optimum protein gathering and function (4). Alterations in the levels of these essential cofactors have been regarded as a mechanism for bypassing the anti-proliferative effects of endocrine therapies (4). The identification of numerous ER-DNA interaction sites has led to the discovery of new ER-related proteins, which participate in the stabilization of the ER complex on the chromatin. FOXA1, GATA3, PBX1, and AP2γ are among the transcription factors involved in this process (4). FOXA1 and GATA3 have been shown to be consistently expressed in the luminal subtype of breast cancer, indicating the presence of a co-modulatory loop that might contribute to the maintenance of the luminal phenotype (5). FOXA1 also enhances response to estrogen through regulating ER binding with the promoter region of its targets (6, 7). GATA3 has a regulatory role in expression of FOXA1, which enhances ER expression in epithelial cells (8). Meanwhile, another transcription factor namely FOXM1 has been demonstrated to decrease expression of GATA3 *via* induction of methylation in its promoter through recruitment of a DNA methyl transferase. As an estrogen-inducible gene (9), FOXM1 also participates in the regulation of the functional interaction between ER and GATA3. Therefore, GATA3, FOXA1, FOXM1 and ER construct an interactive network which directs the fate of cells in the breast tissue (8). Based on the importance of FOXM1/GATA3/FOXA1/ESR1 axis, we designed the current study to find long non-coding RNAs (lncRNAs) that are linked with transcription factors through a systems biology approach. Subsequently, we assessed expression of these genes in breast cancer tissues versus adjacent non-cancerous tissues (ANCTs). LncRNAs have crucial roles in the regulation of expression of genes through various mechanisms including adjustment of the chromatin configuration, recruitment of transcription factors, modulation of stability of transcripts, changing the bioavailability of other biomolecules and interaction with RNAs and proteins (10). Thus, their interaction with cancer related axes including FOXM1/GATA3/FOXA1/ESR1 axis might affect the course of breast cancer.

## Materials and Methods

**Figure 1:**
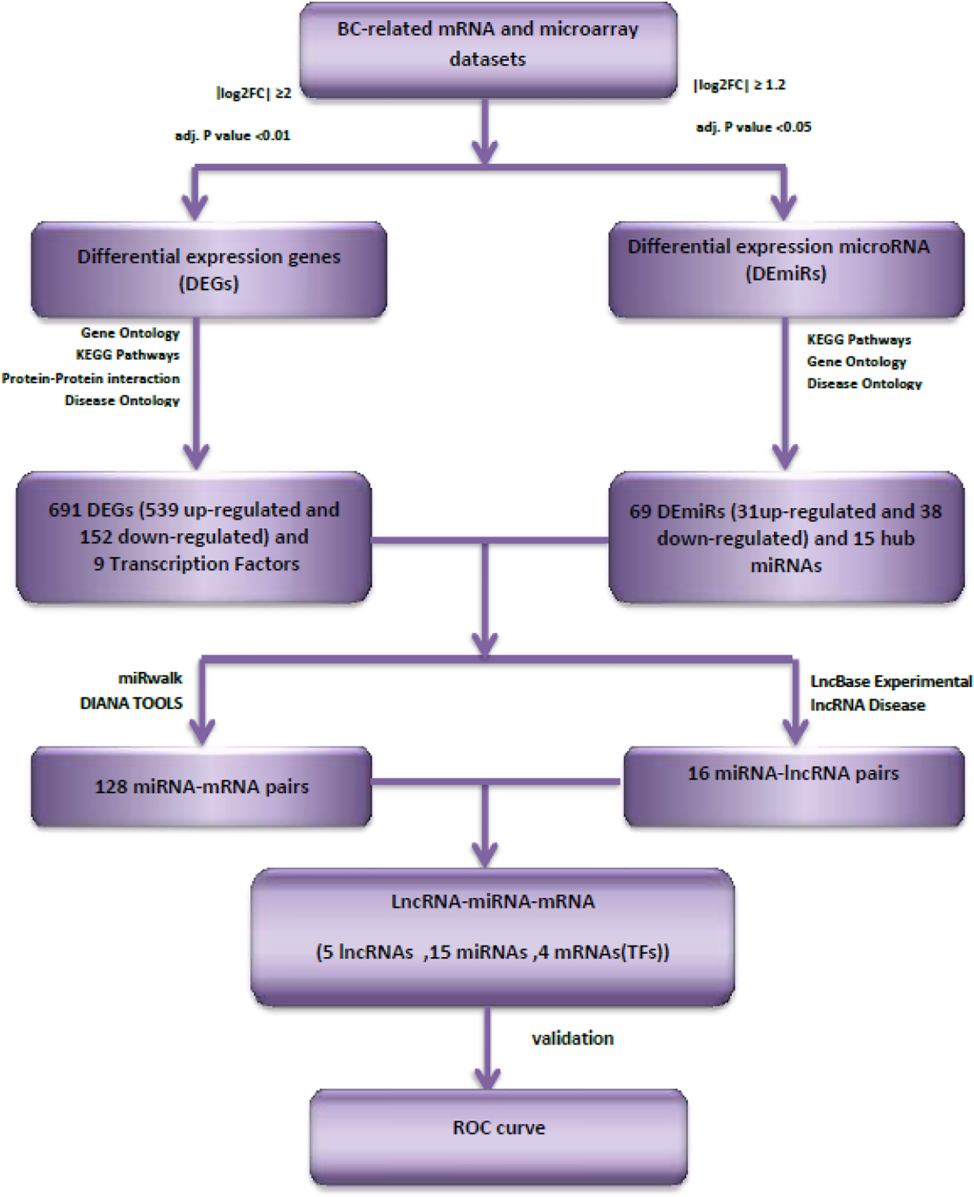
Flowchart of *in silico* assessments.

**Figure 1** shows the overall flowchart of our *in silico* assessments.

### Microarray Gene Expression Data Analysis

The criteria for finding eligible microarray datasets from the Gene Expression Omnibus database (https://www.ncbi.nlm.nih.gov/geo/) were as follows: 1) Inclusion of gene expression data of breast cancer and adjacent normal tissue samples before any patient treatment (Subjects that had received chemotherapy or radiotherapy before surgery were omitted); 2) Arrays having at least 100 tumor and adjacent normal tissue specimens; 3) Inclusion of four molecular subtypes of breast cancer (luminal A, luminal B, Her2-enriched and Triple-negative/basal-like). One dataset was finally included in this study: GSE45827 (11). First, the Affy package in R (12) was used to convert the raw data (.CEL files). Then, the robust multi-array average (RMA) was used to correct and normalize the background. Subsequently, the Bioconductor package in R was used for conversion of the probe information into a gene symbol (13). The average expression value between all probes sets that were mapped with a given gene were used for final gene expression value. Finally, the R package “limma” (14) was utilized to normalize the data and find differentially expressed genes (DEGs). The threshold points were set to adj. P value <0.01 and |log 2-fold-change| ≥2.

### Microarray miRNA Expression Data Analysis

The miRNA microarray datasets GSE59247 (15) and GSE81000 (16) were obtained from GEO database, GPL15019 [Agilent-031181 Unrestricted_Human_miRNA_V16.0_Microarray 030840] and GPL10656 [Agilent-029297 Human miRNA Microarray v14 Rev.2] Platforms, respectively. Differentially expressed miRNAs (DEmiRs) between luminal tumors and normal samples were selected using GEO2R online analysis tool (17). The DEmiRs were obtained with thresholds of |logFC|>1.2 and adj.P value <0.05. Then the common DEmiRs that were in both datasets with the same direction of expression changes (up or down expression in luminal tumors compared with normal samples) were selected.

### Functional Enrichment Analysis

To better comprehend the underlying biological processes and pathways, disease ontology (DO), functional enrichment of gene ontology (GO), Kyoto Encyclopedia of Genes and Genomes (KEGG) pathways analyses were conducted using cluster Profiler, DOSE and in R (18). Statistical significance was reported by the adjusted P-value (Q-value) <0.05.

### Transcription Factors Recognition

TF checkpoint (www.tfcheckpoint.org), a high-quality and comprehensive repository of human, mouse, and rat TF candidates was used to detect TFs among the list of differentially expressed genes (19).

### Protein–Protein Interaction (PPI) Analysis

To conduct a research on protein experimental interactions and prediction, a PPI network was constructed using a STRING online database (http://string-db.org) (combined score >0.4) (20). DEGs with a combined score >0.9 as the threshold (18) were imported into Cytoscape to create network visualization. Then, the network was processed for module analysis, using Molecular Complex Detection (MCODE) Plugin in Cytoscape software with default parameters (21, 22). Parameters were degree cutoff ≥2, node score cutoff ≥2, K-core ≥2, and maximum depth = 100. Cytoscape (v3.8.3) was used to visualize and calculate the basic features of the PPI network, namely average clustering coefficient distribution, closeness centrality, average neighborhood connectivity, node degree distribution, shortest path length distribution, and topological coefficients.

### Construction of the Competing Endogenous RNAs (ceRNAs) Regulatory Network

Based on the potential interaction between mRNAs, miRNAs, and lncRNAs, we also reconstructed a ceRNA regulatory network. First, breast cancer-specific RNAs, including mRNAs and miRNAs, were filtered. Down-/up-regulated mRNAs and miRNAs were assigned using the following parameters: adj.P value <0.01 and |log 2-fold-change| ≥2 and adj.P value <0.05 and |log 2-fold-change| ≥1.2, respectively. Then, the mRNAs that were targeted by DEmiRs were predicted by miRwalk (http://mirwalk.umm.uni-heidelberg.de). If there was no interaction between DEmiRs and hub DEGs, the node was removed. Next, the interactions between lncRNA and miRNA were predicted by DIANA TOOLs databases (https://diana.e-ce.uth.gr/lncbasev3), and a list of lncRNA–miRNA pair interactions was identified. Finally, from this list, the lncRNAs predicted for breast cancer were filtered using the LncRNADisease v3.0 database (http://www.rnanut.net/lncrnadisease). The reconstructed ceRNA network was visualized by Cytoscape (v.3.8.3).

### Patients

A total of 69 breast cancer samples and their corresponding ANCTs were obtained from Iranian female patients who were referred to Farmanieh and Sina hospitals during 2017–2020, Tehran, Iran. The study protocol was approved by the ethical committee of Shahid Beheshti University of Medical Science (ethical code: IR.SBMU.RETECH.REC.1398.379). Patients received no chemotherapy or radiotherapy prior to sample collection. All samples were conveyed in liquid nitrogen to the Department of Medical Genetics and stored in −80°C till succeeding expression analyzes. Patients’ medical records were assessed for collecting related pathological data.

### Expression Assays

All samples were subjected to RNA isolation using the RiboEx Total RNA extraction kit (GeneAll, Seoul, South Korea). Then, 50–100 ng of RNA was transformed to cDNA using the commercial kit (ExcelRT™, SMOBIO, Taiwan). Expressions of genes in tumoral and non-tumoral tissues were measured in the ABI step one plus PCR machine. Expression levels were normalized to transcripts of *B2M*. The commercial real time PCR Master Mix (Ampliqon, Odense, Denmark) was used for preparing the reactions. Information about primers is shown in **Table 1**.

**Table 1:** Sequences of primers used for quantification of genes expressions.

Name	Type	Sequence (5′→3′)	Primer Length	PCR Product (bp)
*ESR1-F*	mRNA	CCTGATGATTGGTCTCGTCTG	21	105
*ESR1-R*		ATGCCCTCTACACATTTTCCC	21	
*FOXA1-F*	mRNA	GGAACAGCTACTACGCAGACA	21	134
*FOXA1-R*		CATGTTGCCGCTCGTAGTCA	20	
*FOXM1-F*	mRNA	CACTGAGAGGAAGCGCATGAC	21	113
*FOXM1-R*		GGTTGTGGCGGATGGAGTTC	20	
*GATA3-F*	mRNA	TCATTAAGCCCAAGCGAA	18	176
*GATA3-R*		CTTCTTCATAGTCAGGGGTC	20	
*ZNF337-AS1-F*	LncRNA	AACCAAACCTACCCACAACG	24	134
*ZNF337-AS1-R*		ACCACTAAGTCAATCCCAGGTG	22	
*APTR -F*	LncRNA	AGGGTGGATGTGCTGTGATGAAGA	20	105
*APTR -R*		AGTCCATAACACCTCCGCAGACAA	23	
*RAMP2-AS1 -F*	LncRNA	TTCATGTGCCAGTCTTCATCTC	22	123
*RAMP2-AS1 -R*		CCATTGACTCTCTCCCACTG	20	
*LINC00663-F*	LncRNA	ACAGCTAGGGACGTGAAAGAA	21	122
*LINC00663-R*		AGGAGCTTATGGAGGTCAGG	20	
*AC144450.1-F*	LncRNA	ACGTAAAGTCCTGGGGACAAG	21	108
*AC144450.1-R*		CATTTCTGTTGACAGTGCGTAG	22	
*B2M-F*	mRNA	AGATGAGTATGCCTGCCGTG	20	105
*B2M-R*		GCGGCATCTTCAAACCTCCA	20	

### Statistical Methods

Statistical analyses were performed using the R programming language. Transcript quantities of nine mentioned genes were calculated in relation to the *B2M* reference gene using the equation:$\frac{amp_{gene}^{-CT_{gene}}}{amp_{B2M}^{-CT_{B2M}}}$. Then, the obtained values were log2 transformed and used for subsequent analysis.

A comparison was made between normal and tumor tissues of patients, and the significance of the difference between mean values was assessed using the paired t-test. Correlations between expressions were evaluated through the calculation of Spearman correlation coefficients. For assessment of the diagnostic power of genes, receiver operating characteristic (ROC) curves were plotted. Three predictive machine learning methods, namely Bayesian Generalized Linear Model, Generalized Linear Model, and Linear Discriminant Analysis with 10-fold cross-validation were used to compute the sensitivity and specificity of each model. The Bayesian Generalized Linear Model (bayesGLM) provided the most efficient estimates, and in the best setting, the AUC was 0.96. Youden’s J statistic was employed to find the optimum threshold. BayesGLM was then selected based on the previous results to investigate the efficiency of each gene for the separation of groups. A Chi-square test was employed to assess the association between patients’ demographic information and transcript levels of genes. Genes with log2FC ≥1 (tumor tissues vs. normal tissues) were considered as up-regulated and those with log2FC ≤−1 were regarded as down-regulated. For all statistical tests, the level of significance was set at P value <0.05.

## Results

### Genes and miRNAs Expression Analyses

After quality control and elimination of inaccurate expression data, we identified a total of 691 DEGs (539 up-regulated and 152 down-regulated) in breast cancer samples compared with control samples with adj.P value <0.01 and |log 2-fold-change| ≥2. Then, 69 DEmiRs (31 up-regulated and 38 down-regulated) with adj.P value <0.05 and |logFC| >1.2 were selected for further analysis.

### PPI Network Reconstruction and Identification of Hubs

**Figure 2:**
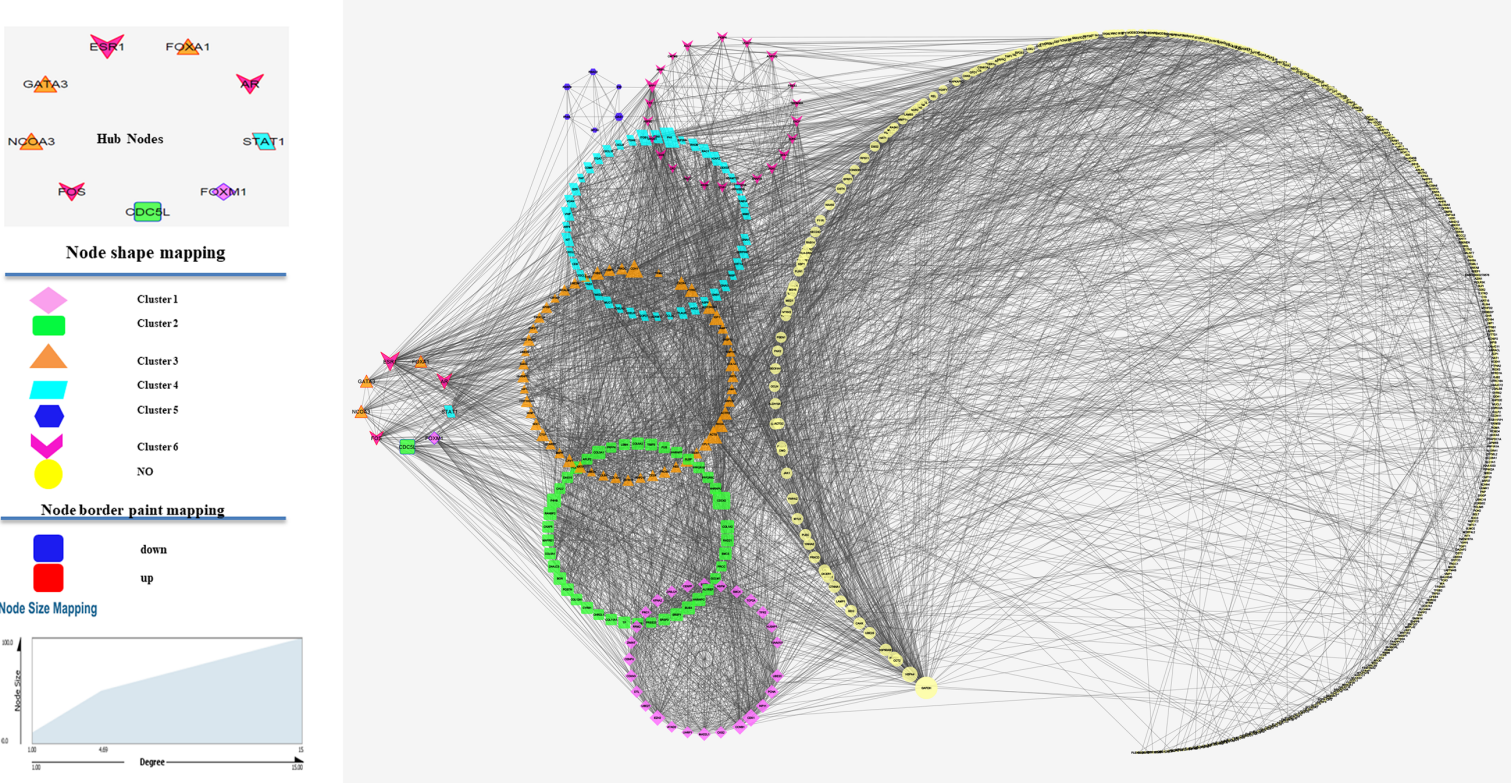
Protein–protein interaction (PPI) analysis of differentially expressed genes. Clusters were identified from the PPI network using the MCODE method with a score of >4.0. Six modules were represented in the figure in unique shapes and colors. Edge stands for the interaction between two genes. The importance of protein nodes in the network is described by degree where small and large sizes denote low and high values, respectively. The border color represents the fold change for nodes where upregulated nodes are marked red and downregulated nodes marked blue. This figure shows the PPI between genes in the selected dataset showing the clusters related to each of key genes.

The STRING database was used to predict the interaction relationship. As a result, 629 nodes and 4,252 protein pairs with a combined weight score >0.4 were found in the network. All nodes with a combined score >0.9 were imported into Cytoscape software for visualization. After clustering analysis with MCODE, six modules with score >4 were detected. In these clusters, the MCODE score for cluster 1 was 26.44, including 28 nodes; the MCODE score for cluster 2 was 10.97, consisting of 42 nodes; the MCODE score for cluster 3 was 9.1, including 53 nodes; the MCODE score for cluster 4 was 6.97, including 44 nodes; the MCODE score for cluster 5 was 5.6, consisting of 6 nodes; the MCODE score for cluster 6 was 4.3, including of 53 nodes. After centrality analysis, the nodes with degree, closeness, and betweenness indices values higher than the mean value of the whole network were considered as hub nodes. All transcription factors were filtered in entire network using TFcheckpoint and 31 TFs were found. We selected 9 of 31 transcription factors based on centrality indices and MCODE results. So, ESR1, AR, FOS, STAT1, NCOA3, GATA3 and FOXA1 in the up-regulated network and CDC5Land FOXM1 in the down-regulated network were considered as candidate nodes. **Figure 2** shows the PPI network of the selected DEGs.

### Functional Annotation

**Figure 3:**
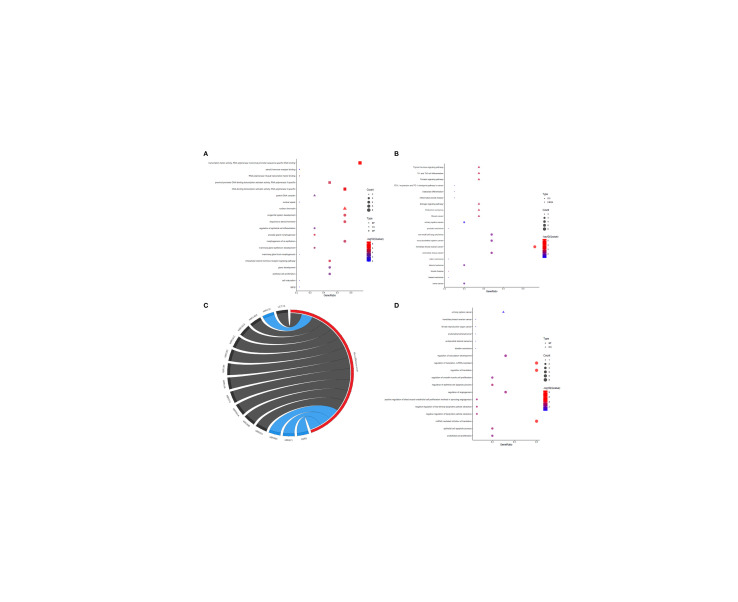
Functional annotation for DEGs and DEmiRs. **(A)** Gene Ontology for DEGs; **(B)** KEGG pathway and Disease Ontology for DEGs; **(C)** KEGG pathway for DEmiRs; **(D)** Gene Ontology and Disease Ontology for DEmiRs. KEGG pathway represented by circulary segment. Each ribbon represents a miRNA. The ribbon color represents the fold change for miRNAs where upregulated ribbons are blue and downregulated ribbons marked black.

To obtain the biofunctions of the TFs, DO functional, GO analyses and KEGG pathway enrichment were conducted using the clusterProfiler package of R software (**Figures 3A, B**). The same method was performed to analyze miRNAs. The result is shown in **Figures 3C, D**. The ESR1 gene encodes ERα, which plays central roles in mammary carcinogenesis and clinical response of breast tumors to endocrine therapy. In our network, ESR1 with greater degree than other hub nodes was selected. After functional enrichment analysis of hub nodes, three transcription factors that interact with ESR1 in luminal breast cancer cells were filtered for further analyses. FOXA1, FOXM1, and GATA3 transcription factors are involved in some common biological pathways such as response to steroid hormone, mammary gland epithelium development, urogenital system development, intracellular steroid hormone receptor signaling pathway, aging and epithelial cell proliferation.

Through function enrichment of the DEmiRs, 15 hub miRNAs were identified for further studies.

### Construction of the lncRNA–miRNA–mRNA Regulatory Network

**Figure 4:**
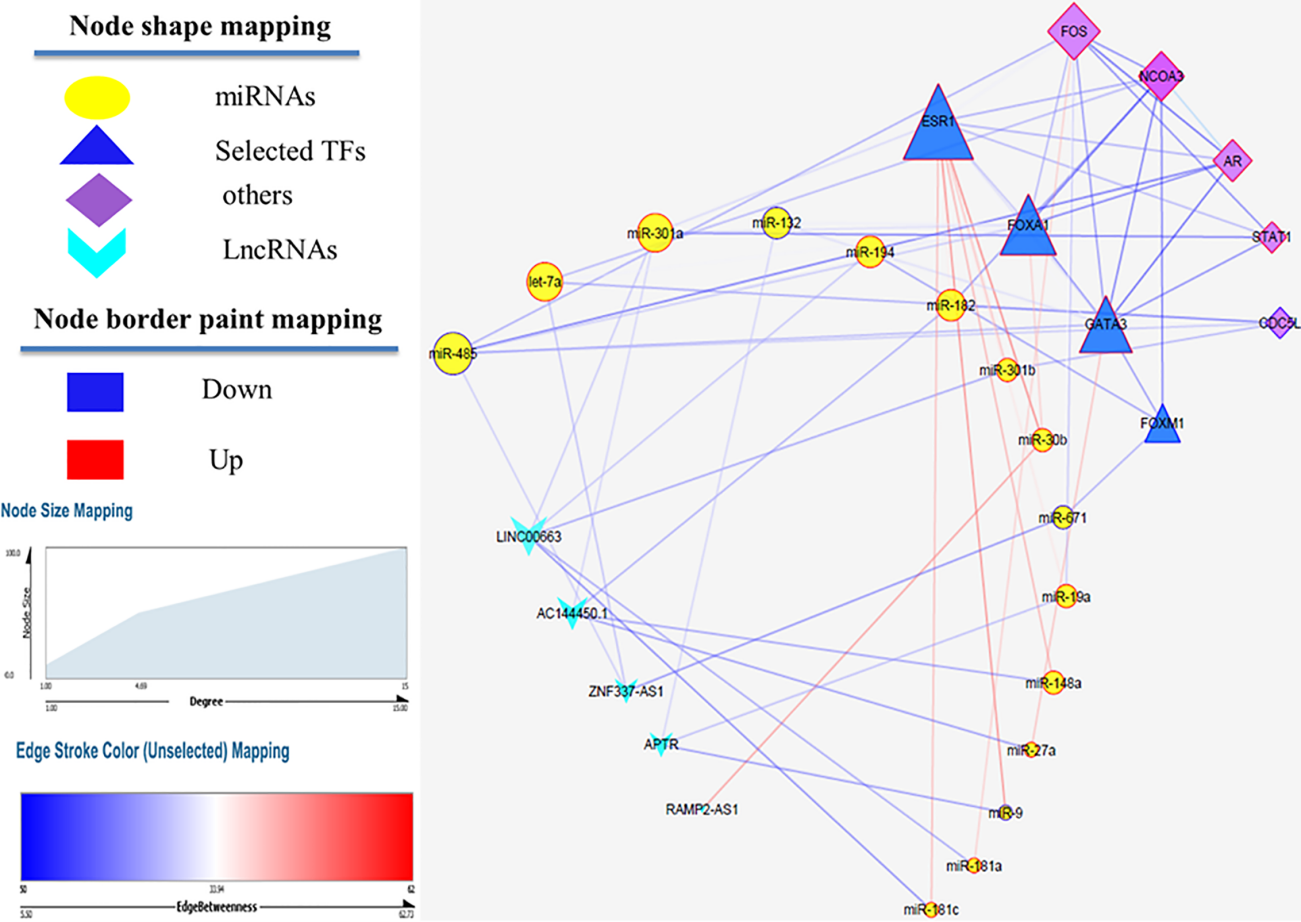
LncRNA–miRNA–mRNA network. Edge stands for the interaction between two genes. The importance of protein nodes in the network is described by degree where small and large sizes denote low and high values, respectively. The border color represents the fold change for nodes where upregulated nodes are marked red and downregulated nodes marked blue.

MiRwalk database was searched to predict the interaction between miRNAs and mRNAs. If the predicted genes from the database were not considered as hub nodes, they were removed from our list. So, 128 miRNA–mRNA interaction pairs were identified using our strategy. After identifying hub miRNAs, among 128 miRNA-hub Gene pairs, hub mirRNAs were filtered and 19 miRNA–mRNA interaction pairs were detected between hub miRNAs and four selected transcription factors. Next, lncRNAs targets of the selected miRNAs were predicted using the DIANA TOOLs database. Then, we determined five lncRNAs with a putative role in breast cancer using the LncRNA disease (v.3.0) database, including AC144450.1, APTR, RAMP2-AS1, LINC00663, and ZNF337-AS1. LncRNAs can act as an endogenous sponge and bind directly to miRNAs, resulting in the down-regulation of miRNAs and up-regulation of gene, i.e., they regulate the regulator. Finally, a lncRNA–miRNA–mRNA regulation network was reconstructed and visualized by Cytoscape (V. 3.8.3) (**Figure 4**). LncRNAs, mRNAs and miRNAs are the nodes in the network. The edges in the network represent the interactions between different types of RNAs. In total, we selected five lncRNAs and four TFs in this triple regulatory network to validate their expressions in clinical samples.

### Demographic Data

The same cohort of patients has been used in our previous study (23). Demographic data of study participants is summarized in **Table 2**.

**Table 2:** Demographic and clinical data of breast cancer patients.

Parameters	Values
Menarche age (mean ± SD)	13.15 ± 1.56 (10–18)
Menopause age (mean ± SD)	49.47 ± 5.08 (38–60)
First pregnancy age (mean ± SD)	21.09 ± 4.69 (14–37)
Breast feeding duration (months) (mean ± SD)	47.85 ± 48.88 (0–240)
Cancer stage (%)	
I	18 (26.08%)
II	22 (31.88%)
III	20 (28.98%)
IV	5 (7.24%)
Unknown	4 (5.79%)
Overall grade (%)	
I	12 (17.39%)
II	32 (46.37%)
III	18 (26.08%)
Unknown	7 (10.14%)
Mitotic rate (%)	
I	23 (33.33%)
II	26 (37.68%)
III	7 (10.14%)
Unknown	13 (18.84%)
Abortion	
Positive	57 (82.60%)
Negative	12 (17.39%)
Oral contraceptive use	
No	35 (50.72%)
Yes	34 (49.27%)
Hormone replacement therapy	
No	58 (84.05%)
Yes	11 (15.94%)
Estrogen receptor	
Positive	52 (75.36%)
Negative	13 (18.84%)
Unknown	4 (5.79%)
Progesterone receptor	
Positive	48 (69.56%)
Negative	14 (20.28%)
Unknown	7 (10.14%)
Her2/neu expression	
Positive	13 (18.84%)
Negative	50 (72.46%)
Unknown	8.69 (7.5%)

### Expression Assays

The expression of *GATA3* was significantly higher in breast cancer tissues compared with ANCTs (Ratio of mean expressions (RME) = 4.99, P value = 3.12E−04). Moreover, expression levels of *APTR*, *AC144450.1*, and *ZNF337.AS1* were elevated in breast cancer tissues compared with control tissues (RME = 2.27, P value = 5.40E−03; Ratio of mean expressions = 615.95, P value = 7.39E−19 and RME = 1.78, P value = 3.40E−02, respectively). On the other hand, the expression of RAMP2.AS1 was lower in breast cancer tissues than controls (RME = 0.31, P value = 1.87E−03). Expression levels of *FOXA1*, *ESR1*, and *FOXM1* and *linc00663* were not significantly different between the two sets of samples. The patients’ cohort included 6 HER2 subtype, 2 TNBC and 62 luminal cases. The observed pattern of expression did not vary between these subtypes. **Table 3** shows the detailed statistics of expressions of named transcription factors and related lncRNAs in breast cancer samples compared with non-cancerous tissues.

**Table 3:** Detailed statistics of expressions of named transcription factors and related lncRNAs in breast cancer samples compared with non-cancerous tissues.

	SE	Ratio of Mean Expressions	P Value	95% CI	
**ESR1**	0.68	1.33	5.44E−01	−0.94	1.77
**FOXA1**	0.44	1.02	9.57E−01	−0.87	0.91
**GATA3**	0.61	4.99	**3.12E**−**04**	1.10	3.53
**FOXM1**	0.64	1.23	6.43E−01	−1.00	1.60
**APTR**	0.41	2.27	**5.40E**−**03**	0.36	2.01
**AC144450.1**	0.66	615.95	**7.39E**−**19**	7.93	10.60
**linc00663**	0.41	0.68	1.85E−01	−1.36	0.27
**ZNF337.AS1**	0.38	1.78	**3.40E**−**02**	0.07	1.60
**RAMP2.AS1**	0.53	0.31	**1.87E**−**03**	−2.76	−0.66

Bold font indicates statistical significance.

**Figure 5:**
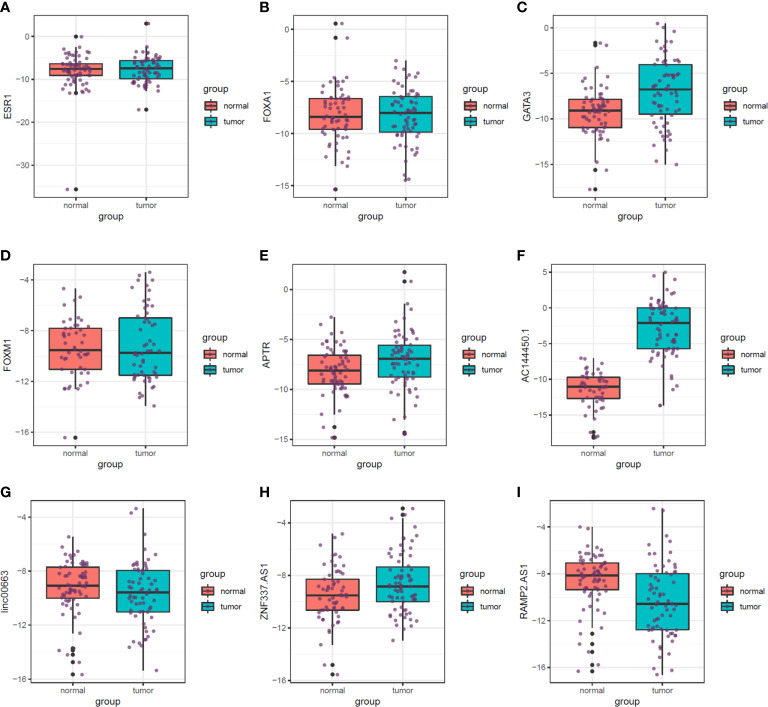
Relative expression of transcription factors and related lncRNAs in breast cancer tissues and their paired normal tissues. Median, upper, and lower quartile values are shown. **(A)** ESR1. **(B)** FOXA1. **(C)** GATA3. **(D)** FOXM1. **(E)** APTR. **(F)** AC144450.1. **(G)** linc00663. **(H)** ZNF337.AS1. **(I)** RAMP2.AS1.

**Figure 5** shows the relative expression of named transcription factors and related lncRNAs in breast cancer tissues and ANCTs.

### Association Between the Expression of Genes and Demographic/Clinical Data

Expression of *GATA3* was significantly associated with the stage (P value = 4.77E−02). Moreover, expressions of *FOXA1* and *RAMP2.AS1* were associated with the mitotic rate (P values = 2.18E−02 and 1.77E−02, respectively). Finally, expressions of *FOXM1* and *ZNF337.AS1* were associated with breast feeding duration (P values = 3.88E−02 and 4.33E−02, respectively). **Table 4** shows the detailed statistics of the association between the expression of genes and demographic/clinical data.

**Table 4:** Association between expression of genes and demographic/clinical data (Log2FC ≤−1 and log2FC ≥1 were regarded as down-regulation and up-regulation, respectively. Same levels of expression were described by −1< log2FC <1).

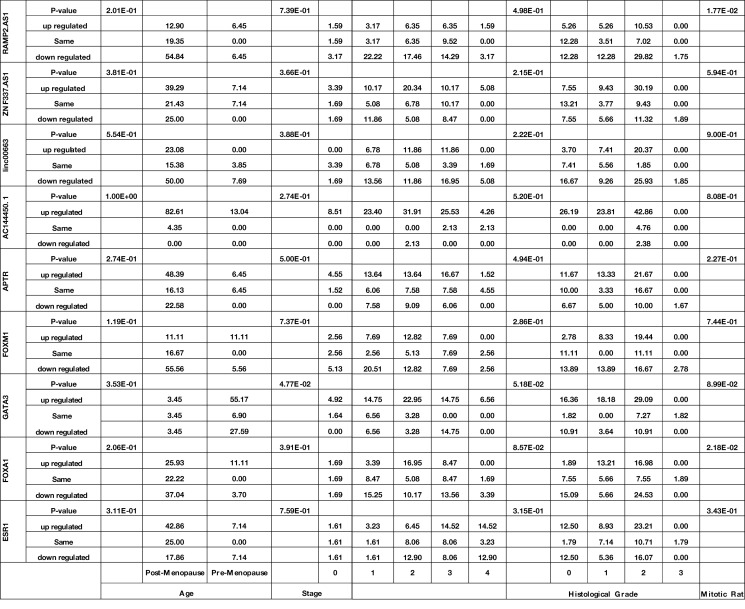
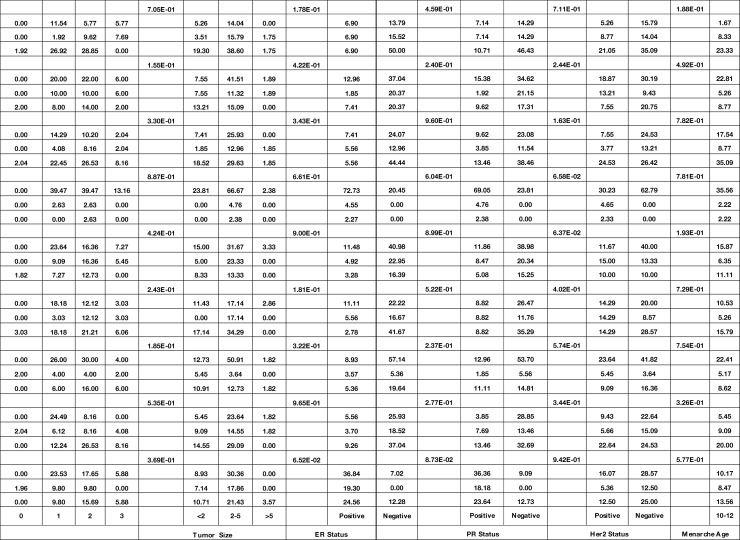
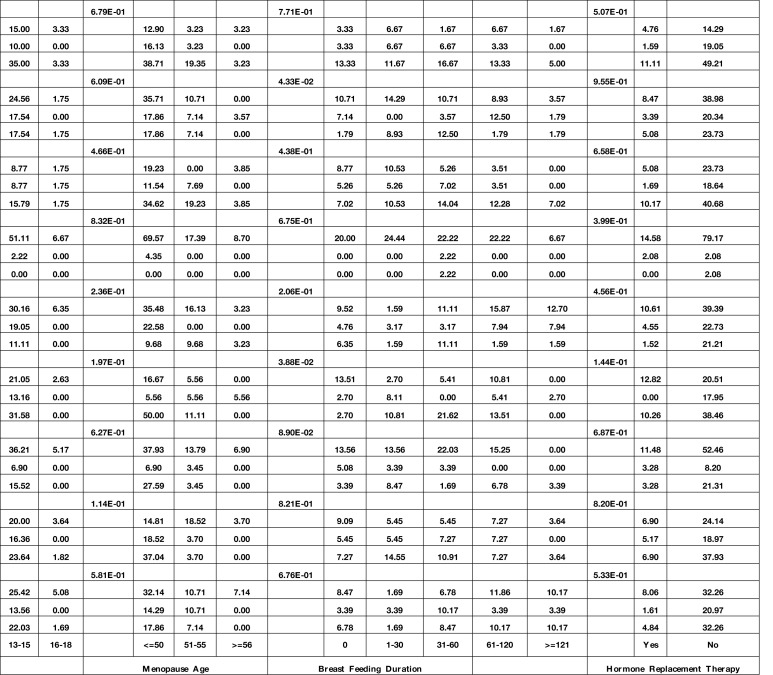

Bold font indicates statistical significance.

### ROC Curve Analysis

**Figure 6:**
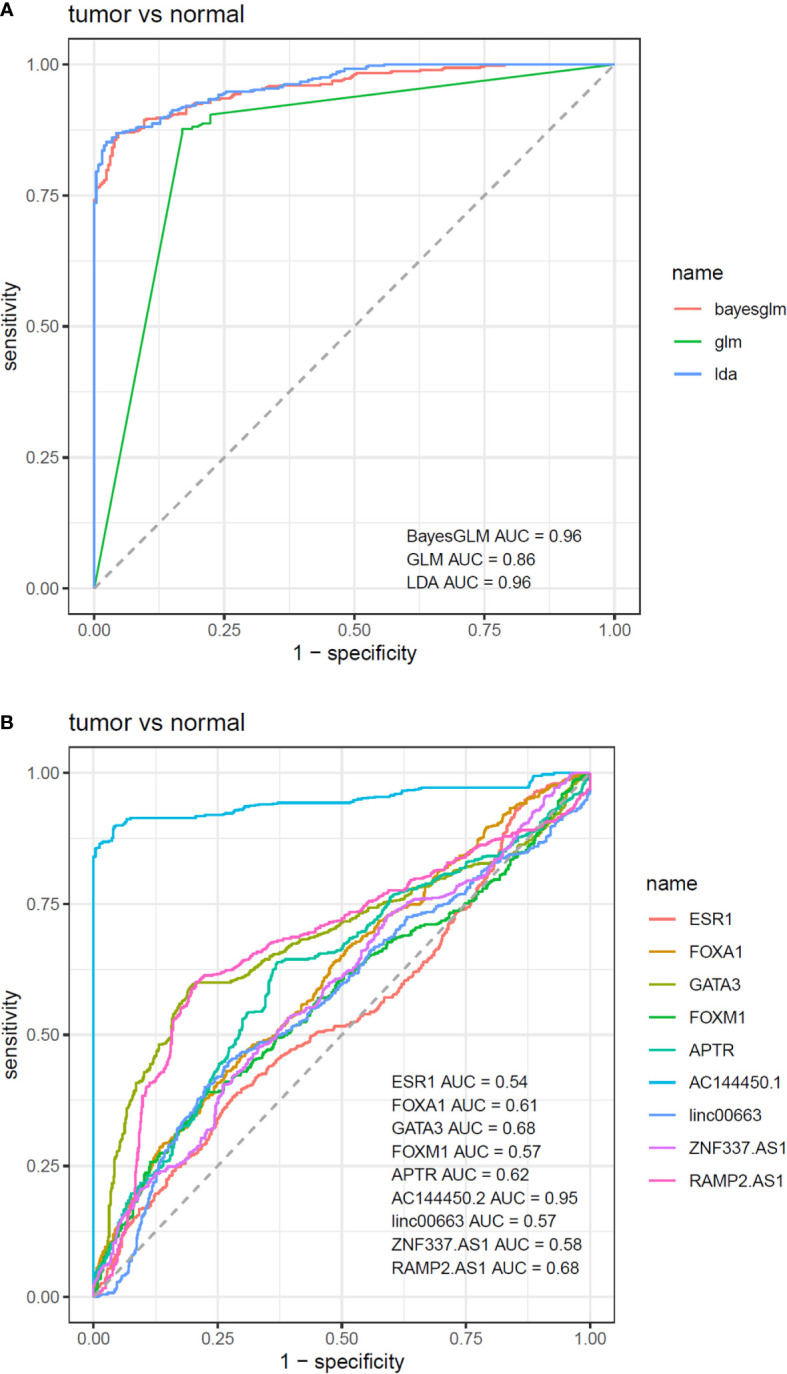
ROC curves depicted by three predictive machine learning methods, namely Bayesian Generalized Linear Model, Generalized Linear Model, and Linear Discriminant Analysis **(A)**. The Bayesian Generalized Linear Model (bayesGLM) provided the most efficient estimates **(B)**.

Based on the AUC values, *AC144450.1* had the optimal diagnostic power in distinguishing between cancerous and non-cancerous tissues (AUC = 0.95, Sensitivity = 0.90, Specificity = 0.96). The combination of expression levels of all genes slightly increased the diagnostic power (AUC = 0.96). **Table 5** and **Figure 6** show the details of the ROC curve analysis.

**Table 5:** Details of ROC curve analysis.

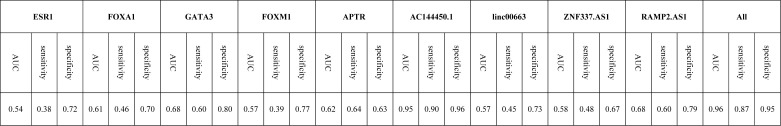

### Correlation Analysis

**Figure 7:**
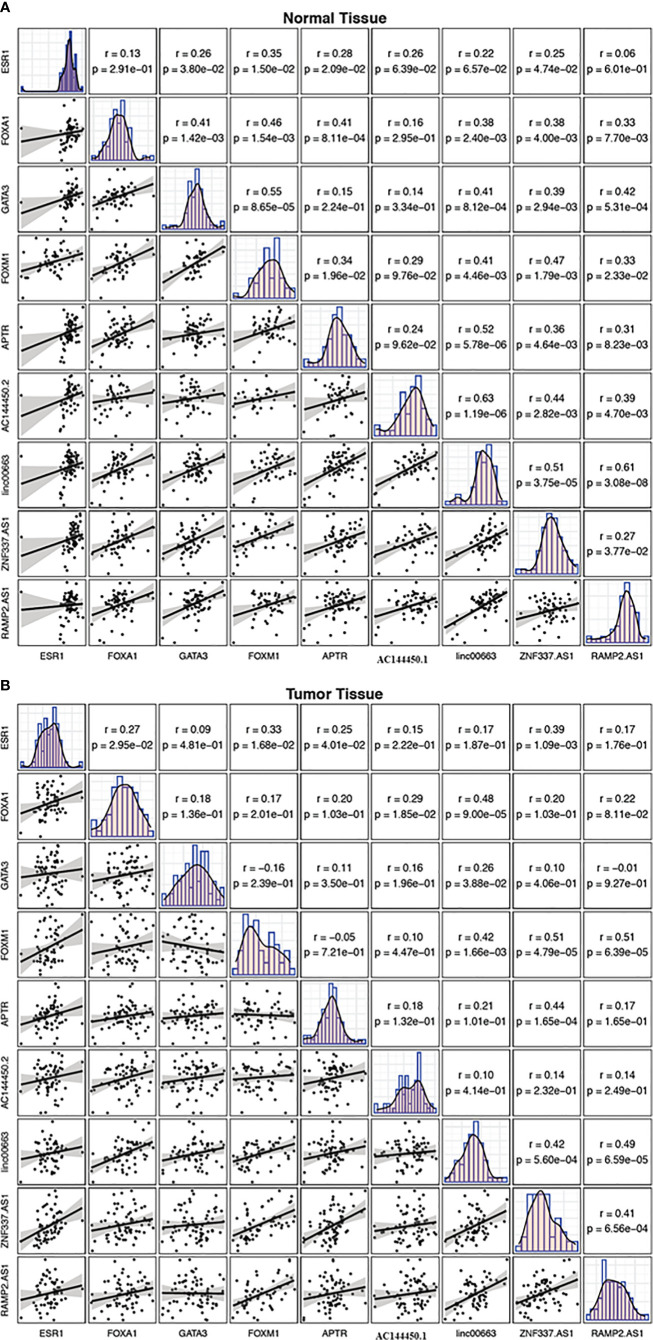
Correlation matrix showing the correlation between expression levels of *FOXM1*, *GATA3*, *FOXA1*, *ESR1* and related lncRNAs in non-cancerous tissues **(A)** and breast cancer tissues **(B)**. The distribution of each variable is shown on the diagonal. The bivariate scatter plots with a fitted line are presented at the bottom of the diagonal. The correlation coefficients and P values are presented on top of the diagonal.

While there were several substantial pairwise correlations between expression levels of genes in non-tumoral tissues, the most robust correlation was noticed between *linc00663* and *RAMP2.AS1* (r = 0.61, P value = 3.08E−8). In the breast cancer tissues, the most strong correlations were reported between *FOXM1*/*ZNF337.AS1* and *FOXM1*/*RAMP2.AS1* pairs (r = 0.51, P value = 4.79E−5 and r = 0.51, P value = 6.39E−5, respectively). **Figure 7** shows the correlation matrix between expression levels of named transcription factors and related lncRNAs in two sets of samples.

## Discussion

**Figure 8:**
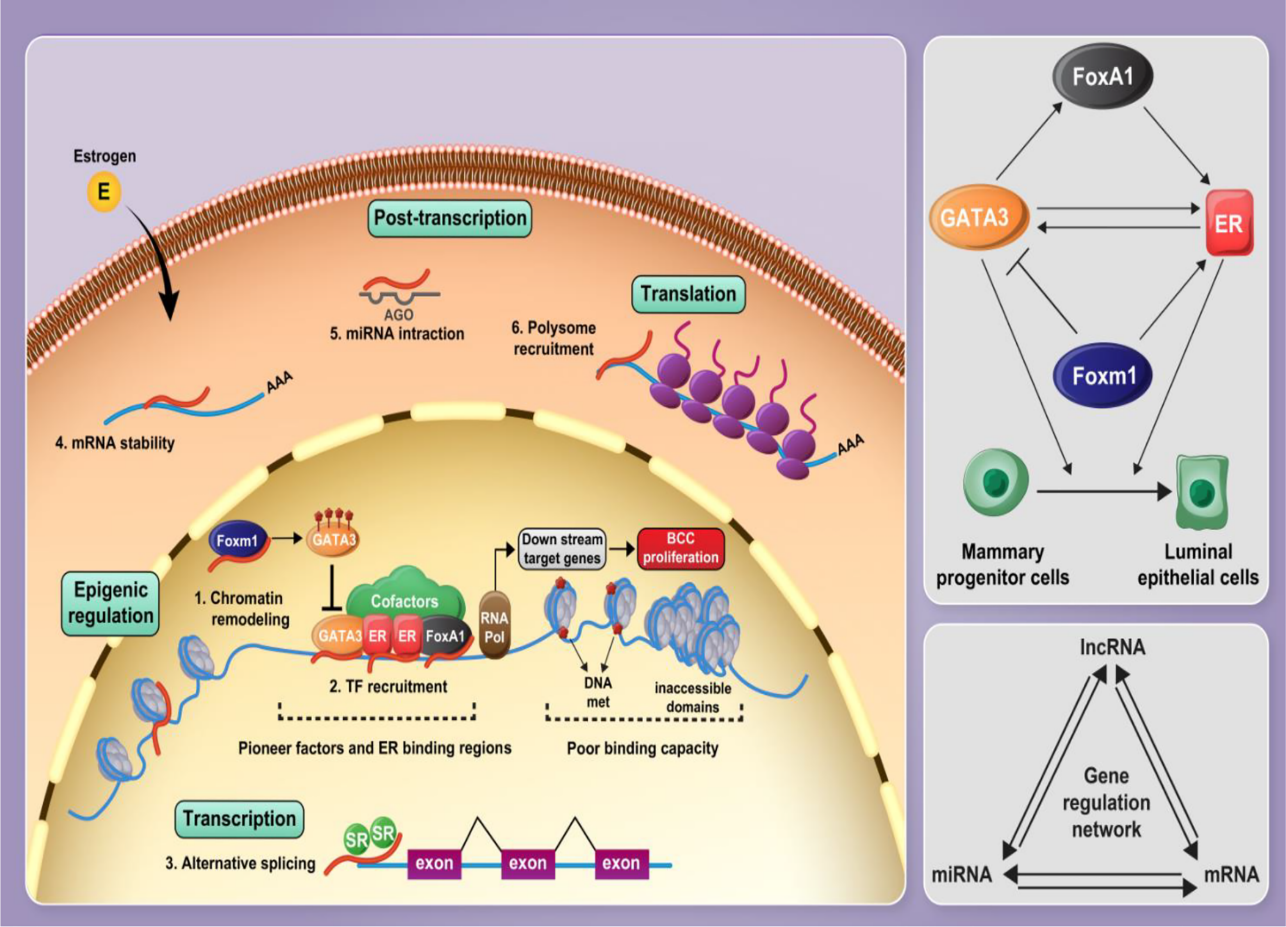
Several cofactors along with pioneer factors including FOXA1, ER1, and GATA3 contribute to establish a complex on the chromatin, modulating E2 signaling and mammary progenitor cell fate and promoting their differentiation into luminal epithelial cells. FOXA1 facilitates euchromatic conditions for ER through the formation of a stable platform for the recruitment of ER-associated coregulators and mediating chromatin loops with a subset of target gene promoters. GATA3 and ER are involved in a positive feedback regulation loop. FOXM1 regulates GATA3 expression by promoting DNA methylation leading to the expansion of mammary stem and progenitor pools. Non-coding RNAs, particularly lncRNAs and miRNAs, play critical roles in the regulation of protein-coding genes and, along with mRNAs, form a gene expression regulatory network. Gene expression regulation is influenced by lncRNAs at different levels, such as epigenetic, transcriptional level, post-transcriptional processes, and translational level.

In the current project, we developed an *in silico* approach to identify lncRNAs, which are functionally associated with *FOXM1*, *GATA3*, *FOXA1* and *ESR1*. Subsequently, we validated the results of *in silico* methods in clinical samples and assessed the expression of these genes and five lncRNAs in breast cancer samples and ANCTs. **Figure 8** shows the interactions between the selected mRNAs and role of lncRNAs in the regulation of protein-coding genes.

Expression analysis of the mentioned genes in a cohort of breast cancer patients revealed overexpression of *GATA3* in breast cancer tissues compared with ANCTs. This transcription factor has an essential role in the development of the normal mammary gland. In addition, it is regarded as the amplest transcription factor in luminal epithelial cells (24). The role of GATA3 as an oncogene or tumor suppressor gene is a matter of debate. The clustering of identified *GATA3* mutations within specific functional protein domains is in line with the suggested description of an oncogene (24). Cimino-Mathews et al. have assessed GATA3 expression in a set of invasive ductal carcinomas samples as well as matched metastatic breast carcinomas using the tissue microarray method. They reported GATA3 labeling in two-thirds of primary ductal carcinomas, including triple-negative samples. Remarkably, GATA3 labeling was preserved in paired metastases and all “luminal loss” metastases that have lost ER and/or PR expression, indicating the application of this marker for diagnosis of metastatic breast cancers (25). Although several studies have shown an association between GATA3 and ER signaling pathway (2, 26, 27), the observed preservation of GATA3 expression in primary triple-negative breast cancer samples and metastatic tissues of luminal cancers in the absence of ER expression suggests dissociation of GATA3 from ER signaling in at least some cases of breast cancer (25). Consistent with this speculation, we could not find any association between *GATA3* expression and ER status. In turn, we detected a significant association between expression levels of this gene and breast cancer stage. McCleskey et al. have demonstrated the significance of GATA3 expression in the determination of patients’ prognosis. Yet, they reported a similar expression of this protein between advanced luminal tumors and early-stage luminal tumors (28). The reason for inconsistency between our results and the results of the mentioned study is the difference in the expression assay technique (Immunohistochemical and *In Situ* Hybridization versus real-time PCR). Notably, correlation analysis revealed a significant correlation between expression levels of *ESR1* and *GATA3* in non-cancerous tissues despite lack of correlation in tumor tissues, suggesting the impact of cancer development on the functional association between these two genes.

Moreover, expression levels of *APTR*, *AC144450.1*, and *ZNF337.AS1* were elevated in breast cancer tissues compared with control tissues. *APTR* has been previously shown to participate in the progression of osteosarcoma *via* inhibition of miR-132-3p expression and over-expression of yes-associated protein 1 (29). This miRNA also represses the progression of breast cancer (29). Therefore, in addition to the connection between *APTR* and ER signaling, over-expression of this lncRNA in breast cancer cells might facilitate the development of this cancer *via* repression of miR-132-3p expression. Moreover, this lncRNA has functional association with CDKN1A/p21 promoter and suppresses its expression through recruitment of polycomb proteins (30). Over-expression of p21 can preclude growth of ERα-positive breast cancer cells (31). Therefore, *APTR* might contribute in the pathogenesis of breast cancer *via* different mechanisms. Although the exact mechanism of *AC144450.1* in the pathogenesis of cancer is unknown, Zhao et al. have reported over-expression of the fusion transcript *PXDN*-*AC144450.2* in prostate cancer samples (32). This lncRNA has been among the differentially expressed lncRNAs between ductal carcinoma *in situ* and early stage breast cancer samples (33).

On the other hand, the expression of *RAMP2.AS1* was lower in breast cancer tissues compared with controls. This lncRNA has been suggested to have a tumor-suppressive impact in glioblastoma *via* an indirect suppression of NOTCH3 (34). NOTCH3 expression contributes in the development of metastasis in both ERα positive and triple-negative breast cancer models (35). Therefore, *RAMP2.AS1* might affect breast cancer pathogenesis *via* both ESR-dependent and -independent manners.

Combination of bioinformatics approaches and literature review has led to suggestion of some putative functional axes in breast cancer. Expression of none of these lncRNAs has been assessed in breast cancer. Based on the previous reports, a number of miRNAs have been identified that might mediate the regulatory role of these lncRNAs. APTR/miR‐132‐3p/YAP1 is a possible functional axis participating in the pathogenesis of osteosarcoma (36). Consistent with this study, in the bioinformatics steps of our study, we have identified miR-132-3p as one of differentially expressed miRNAs between breast cancer samples and non-cancerous samples. Additionally, miR-132-3p has been shown to bind with 3’ UTR of *FOXA1* and suppress its expression, thus reducing breast cancer cells proliferation (37). Therefore, it is possible that *APTR* over-expression leads to down-regulation of miR-132-3p and release FOXA1 from its inhibitory effects. Thus, *APTR*/miR132-3P/FOXA1 axis possibly contributes in the pathogenesis of breast cancer. GATA3 is another target genes of miR-132-3p based on miRTArbase and miRWalk databases, therefore *APTR*/miR132-3p/GATA3 is another possible route of participation of *APTR* in breast cancer. The observed over-expression of *GATA3* in the current study is consistent with this speculation. miR-9-5p is another differentially expressed miRNA based on our bioinformatics analyses. Down-regulation of miR-9-5p in ER-positive breast cancer cells has been associated with over-expression of *ESR1* (38). It is possible that *APTR* up-regulation affects *ESR1* expression through miR-9-5p. Based on the observed correlation between *APTR* and *ESR1* in the current study, *APTR*/miR-9/ESR1 is another putative functional axis in breast cancer.

*AC144450.1* has functional interactions with miR-182-5p, miR-301a-3p, miR-27a-3p and miR-148a-3p. Notably, *GATA3*, *ESR1* and *FOXA1* are targets for these miRNAs. Besides, *ZNF337-AS1* has functional interactions with miR-671-5p and miR-485. Based on the reported data in miRWalk, *FOXA1* and *GATA3* are putative targets of miR-485. This miRNA has been down-regulated in T47D cells and has a potential tumor suppressor role (39). In addition, miR-485 decreases expression of *PGC-1α*, thus reducing metastatic potential of breast cancer (40). As *GATA3* is a target of this miRNA, it is possible that *ZNF-337-AS1* affects expression of *GATA3 via* this miRNA. *ZNF-337-AS1*/miR-485/FOXA1 and *ZNF-337-AS1*/miR-485/GATA3 are other putative functional axes in the pathogenesis of breast cancer. Finally, miR-30b-5p is a predicted target for *RAMP2.AS1*. *ESR1* and *FOXA1* are targeted by miR-30b-5p. Thus, *RAMP2.AS1*/miR-30b-5p/ESR1 and *RAMP2.AS1*/miR-30b-5p/FOXA1 axes might be involved in the pathogenesis of this kind of cancer.

Altered expressions of these lncRNAs in breast cancer samples have both diagnostic and therapeutic significance. Several therapeutic strategies have been designed to affect the hormone receptor-related pathways in breast cancer. However, response of patients to these options is variable (41). One can suppose that alterations in the regulatory mechanisms of these pathways might affect response of patients to these treatment modalities. Thus, prior identification of expression levels of mentioned lncRNAs in breast cancer samples may predict response of patients. Moreover, the difference in the survival rate of patients with breast cancer (42) necessitates identification of factors which have crucial role in the development of this kind of cancer. As lncRNAs can regulate expression of genes and activity of signaling pathways *via* different routes (43), they are appropriate candidates in diagnostic, prognostic and therapeutic approaches. Assessment of lncRNAs signatures in breast cancer, particularly those related with markers used for molecular subtyping is a practical method for understanding the mechanism of different responses of patients to targeted therapies. LncRNA signatures have been previously used as predictors of survival of breast cancer patients. For instance, a signature of eight lncRNAs associated with immune responses could predict survival of breast cancer patients (44). Moreover, a four lncRNA signature including PVT1, MAPT-AS1, LINC00667 and LINC00938 could precisely divide breast cancer patients into high- and low-risk groups (45).

A number of ER-associated cofactors might be used as markers or as potential therapeutic targets in breast cancer. For instance, deletion of NCoA-1 in animal models has attenuated the growth of tissues in response to steroid hormones (46). The steroid receptor RNA activator SRA which interacts with and regulates the AF-1 domain of ER (47), has been suggested as a prognostic biomarker for a subset of ER-positive/node-negative breast cancer patients (48). These observations potentiate ER-associated coregulators as markers for breast cancer.

Expression levels of *FOXA1*, *ESR1*, and *FOXM1* and *linc00663* at mRNA levels were not significantly different between two sets of samples. Thus, the previously reported dysregulation of these factors might be due to regulatory mechanisms at posttranscriptional level. Expressions of *FOXA1* and *RAMP2.AS1* were associated with mitotic rate, indicating the role of these genes in the carcinogenic processes. Finally, expressions of *FOXM1* and *ZNF337.AS1* were associated with breastfeeding duration. Future studies are needed to elaborate on the mechanisms of such observed associations.

Based on the area under receiver operating characteristics curves, *AC144450.1* had the optimal diagnostic power in distinguishing between cancerous and non-cancerous tissues. The combination of expression levels of all genes slightly increased the diagnostic capability. Therefore, *AC144450.1* can be regarded as a diagnostic biomarker for breast cancer. Based on the heterogeneity of expression levels of lncRNAs among breast cancer patients, a diagnostic panel for this kind of cancer should contain multiple genes whose signatures differentiate several types of neoplastic tissues with different levels of genes expressions. Applicability of any putative diagnostic panel should be assessed in independent samples.

While there were several significant pairwise correlations between expression levels of genes in non-tumoral tissues, the most robust correlation was detected between *linc00663* and *RAMP2.AS1*. In the breast cancer tissues, the strongest correlations were reported between *FOXM1*/*ZNF337.AS1* and *FOXM1*/*RAMP2.AS1* pairs. Although the functional role of these pairs in the progression of breast cancer has not clarified, the altered correlation patterns between these genes in the context of cancer suggest the impact of malignancy on the determination of functional networks among genes.

In brief, the altered expression pattern of FOXM1/GATA3/FOXA1/ESR1-associated lncRNAs in breast cancer suggests future assessment of the functional role of these genes in the development of breast neoplasms. We state using only one gene expression microarray for selection of genes, lack of comparison of expression of genes between luminal, HER2+ and TNBC samples and lack of validation in an independent set of tumor/adjacent normal specimens as limitations of our study which should be addressed in future studies.

## Data Availability Statement

The authors acknowledge that the data presented in this study must be deposited and made publicly available in an acceptable repository, prior to publication. Frontiers cannot accept a article that does not adhere to our open data policies.

## Ethics Statement

The study protocol was approved by the ethical committee of Shahid Beheshti University of Medical Science (ethical code: IR.SBMU.RETECH.REC.1398.379). The patients/participants provided their written informed consent to participate in this study.

## Author Contributions

MT and SG-F supervised the study, wrote the draft, and edited the submission. BH, AS, and AZ performed the experiment. FP and YA analyzed the data and performed the bioinformatic analysis. All authors contributed to the article and approved the submitted version.

## Funding

The current study was supported by a grant from Shahid Beheshti University of Medical Sciences (Grant number: 25813).

## Conflict of Interest

The authors declare that the research was conducted in the absence of any commercial or financial relationships that could be construed as a potential conflict of interest.
